# Crystal structure of hexa­kis­(dimethyl sulfoxide-κ*O*)manganese(II) tetra­iodide

**DOI:** 10.1107/S2056989016017904

**Published:** 2016-11-15

**Authors:** Md Azimul Haque, Bambar Davaasuren, Alexander Rothenberger, Tom Wu

**Affiliations:** a Physical Sciences and Engineering Division, King Abdullah University of Science and Technology, KAUST, Thuwal 23955-6900, Kingdom of Saudi Arabia

**Keywords:** crystal structure, tetra­iodide, octa­hedral coordination, isotypism

## Abstract

The title salt, [Mn(C_2_H_6_OS)_6_]I_4_, is made up from discrete [Mn(DMSO)_6_]^2+^ (DMSO is dimethyl sulfoxide) units connected through non-classical hydrogen bonds to linear I_4_^2−^ tetra­iodide anions. The Mn^II^ ion in the cation, situated on a position with site symmetry -3., is octa­hedrally coordinated by O atoms of the DMSO mol­ecule with an Mn—O distance of 2.1808 (12) Å. The I_4_^2−^ anion contains a neutral I_2_ mol­ecule weakly coordinated by two iodide ions, forming a linear centrosymmetric tetra­iodide anion. The title compound is isotypic with the Co, Ni, Cu, and Zn analogues.

## Abstract 2

The crystal structure of hexa­kis­(dimethyl sulfoxide-κ*O*)manganese(II) tetra­iodide is isotypic with the Co, Ni, Cu and Zn analogues.

## Chemical context   

Inorganic–organic hybrid compounds have attracted significant attention owing to their fascinating structural, optical and electrical properties (Stranks & Snaith, 2015 ▸). In particular, CH_3_NH_3_Pb*X*_3_ hybrids obtained from Pb*X*_2_ and CH_3_NH_3_ *X* (*X* = I, Br, Cl) are inter­esting due to their high absorption coefficient and applications in optoelectronics (Stoumpos & Kanatzidis, 2015 ▸). In general, this family of materials adopts the perovskite *ABX*_3_ structure type, where *A* is an organic cation, which is surrounded by twelve nearest *X* halide anions, and *B* is a metal cation (Grätzel, 2014 ▸). There are continuous efforts on replacing Pb in these hybrids due to its toxicity (Wang *et al.*, 2015 ▸). In the present work, one such attempt was made to produce a hybrid between CH_3_NH_3_I and MnI_2_. However, we obtained instead the title salt [Mn(DMSO)_6_]I_4_ (DMSO is dimethyl sulfoxide), and report here its crystal structure.
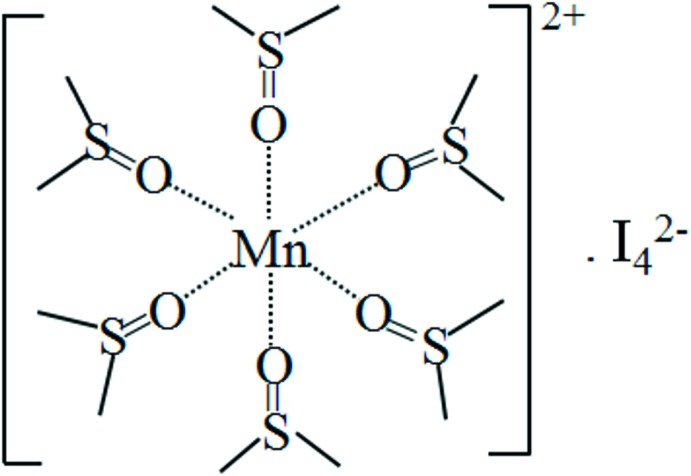


## Structural commentary   

**Figure 1:**
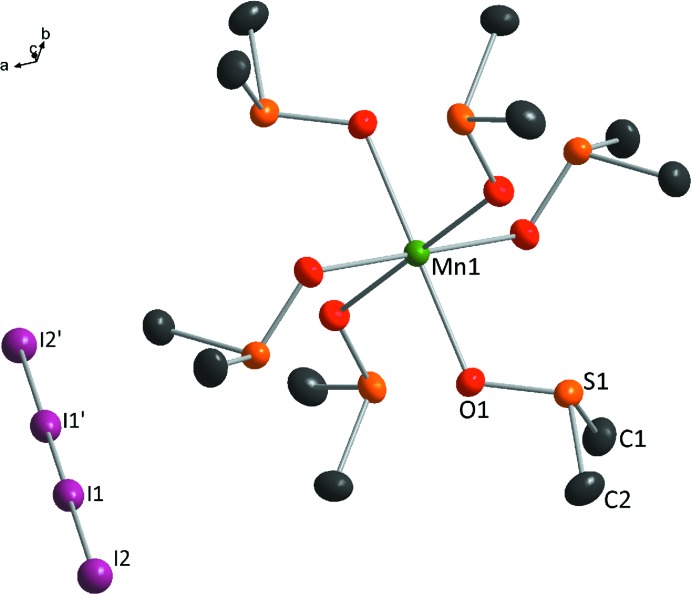
The mol­ecular components of the title compound, with displacement ellipsoids drawn at the 50% probability level. H atoms have been omitted for clarity. [Symmetry code: (′) 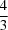 − *x*, 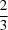 − *y*, 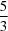 − *z*.]

The title salt is the first compound with a [Mn(DMSO]^2+^ cation and a linear tetra­iodide anion. The Mn^2+^ cation is bound to the O atoms of six DMSO mol­ecules arranged in an octa­hedral configuration (Fig. 1 ▸). Owing to the 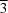. site symmetry of the metal cation, the deviations of corresponding angles from ideal values are minute [range *cis* O—Mn—O angles 86.28 (4)–93.73 (4)°; all *trans* angles 180°]. The Mn—O bond length is 2.1808 (12) Å. The four I atoms are arranged in a linear fashion. The bond length between the two central I atoms is 2.8460 (5) Å; this inner I_2_ moiety is rather weakly bonded to two terminal I^−^ anions with a bond lengths of 3.3251 (6) Å. This confirms the existence of a linear, centrosymmetric polyiodide ion I_4_^2−^, consistent with previous reports (Long *et al.*, 1999 ▸). Both inner and terminal bond lengths of the I_4_^2−^ anion are comparable with values found in [Cu(NH_3_)_4_]I_4_ (Dubler & Linowsky, 1975 ▸) or other [*M*(DMSO)_6_]I_4_ compounds (Long *et al.*, 1999 ▸; Tkachev *et al.*, 1994 ▸; Garzón-Tovar *et al.*, 2013*a* ▸,*b* ▸).

## Supra­molecular features   

**Figure 2:**
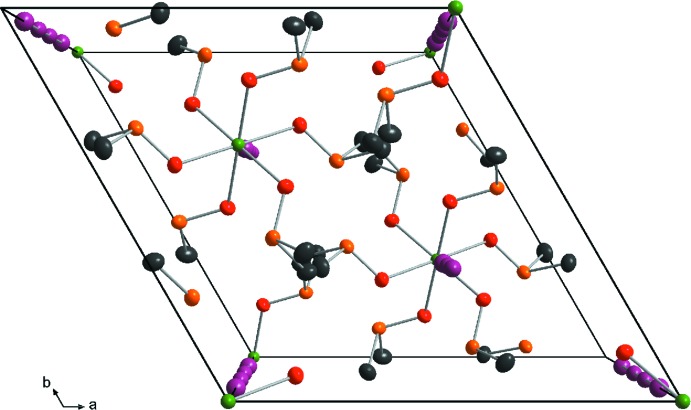
The unit cell of [Mn(DMSO)_6_]I_4_ in a view approximately along [001]. H atoms have been omitted for clarity.

**Figure 3:**
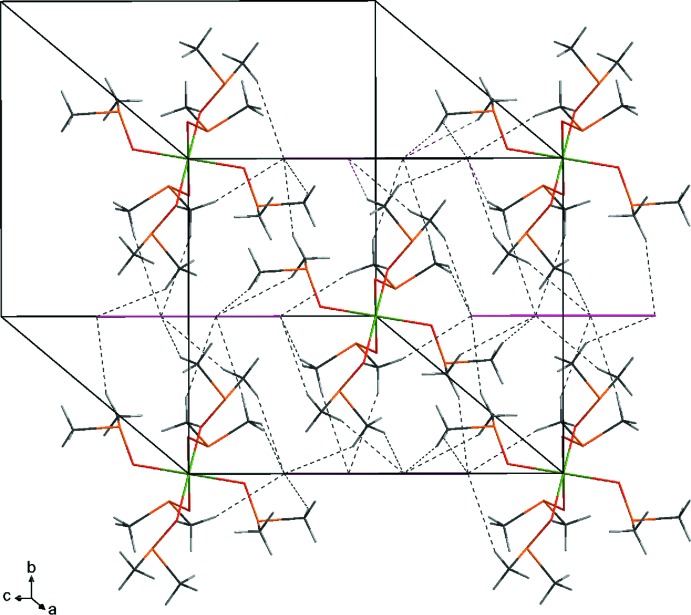
Packing diagram of the title compound. Non-classical hydrogen bonds are shown as dashed lines.

Fig. 2 ▸ shows the unit-cell projection along [001]. The hexa­gonal rod packing of isolated [Mn(DMSO)_6_]^2+^ mol­ecules can be seen along [211]. The tetra­iodide counter-anions are located between the rows (Fig. 3 ▸). An extended three-dimensional supra­molecular network is accomplished through non-classical hydrogen bonding between H atoms of the DMSO mol­ecules and the linear I_4_^2−^ polyiodide anions. Table 1 ▸ collates numerical details of these C—H⋯I inter­actions.

## Database survey   

A number of transition metals have been reported to form complexes with DMSO (Meek *et al.*, 1960 ▸). However, reports on Mn complexes of DMSO with halide anions are scarce, as revealed by a search in the Cambridge Structural Database (Groom *et al.* 2016 ▸). Recently Glatz *et al.* (2016 ▸) reported the crystal structure of [Mn(DMSO)_6_]I_2_. In particular, polyiodide salts are inter­esting compounds owing to their high conductivity and non-linear properties that are predominantly observed in sulfur-rich compounds (Long *et al.*, 1999 ▸). The structure of the title compound is isotypic with the Co, Ni, Cu, and Zn analogues: [Co(DMSO)_6_]I_4_ (Tkachev *et al.*, 1994 ▸), [Ni(DMSO)_6_]I_4_ (Long *et al.*, 1999 ▸), [Cu(DMSO)_6_]I_4_ (Garzón-Tovar *et al.*, 2013*a* ▸), [Zn(DMSO)_6_]I_4_ (Garzón-Tovar *et al.*, 2013*b* ▸).

## Synthesis and crystallization   

The title manganese salt was formed in the course of the targeted synthesis of a hybrid compound between CH_3_NH_3_I and MnI_2_. Anhydrous MnI_2_ and dimethyl sulfoxide (DMSO) were purchased from Alfa–Aesar and Sigma–Aldrich, respectively. CH_3_NH_3_I was purchased from Dyesol. The precursors were used without further purification. The title manganese salt was synthesized by adding anhydrous MnI_2_ (308.7 mg) and CH_3_NH_3_I (158.9 mg) in a glass vial. Then 2 ml DMSO was added to the vial (capped afterwards) and stirred at 353 K for 24 h inside a nitro­gen glove box. A reddish-black solution was observed after 24 h which was cooled down to room temperature and left for 7 d undisturbed. Single crystals of the title compound were obtained as the only solid product after 7 d. The crystals were removed from the vial and dried under nitro­gen flow.

## Refinement   

Crystal data, data collection and structure refinement details are summarized in Table 2 ▸. The methyl H atoms were treated as riding and *U*_iso_(H) values set at 1.5*U*_eq_(C).

## Supplementary Material

Crystal structure: contains datablock(s) I. DOI: 10.1107/S2056989016017904/wm5337sup1.cif

Structure factors: contains datablock(s) I. DOI: 10.1107/S2056989016017904/wm5337Isup2.hkl

CCDC reference: 1515632

Additional supporting information: crystallographic information; 3D view; checkCIF report

## Figures and Tables

**Table 1:** Hydrogen-bond geometry (Å, °)

*D*—H⋯*A*	*D*—H	H⋯*A*	*D*⋯*A*	*D*—H⋯*A*
C1—H1*A*⋯I2	0.98	3.10	3.954 (2)	147
C2—H2*B*⋯I1^i^	0.98	3.26	4.182 (2)	158
C2—H2*C*⋯I2^ii^	0.98	3.20	4.108 (2)	154

Symmetry codes: (i) 
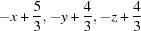
; (ii) 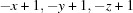.

**Table 2:** Experimental details

Crystal data	
Chemical formula	[Mn(C_2_H_6_OS)_6_]I_4_
*M*_r_	1031.31
Crystal system, space group	Trigonal, *R* 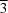
Temperature (K)	200
*a*, *c* (Å)	11.8702 (10), 19.3860 (18)
*V* (Å^3^)	2365.6 (5)
*Z*	3
Radiation type	Mo *K*α
μ (mm^−1^)	4.75
Crystal size (mm)	0.16 × 0.12 × 0.05

Data collection	
Diffractometer	Stoe IPDS2
Absorption correction	Numerical (*X-RED32*; Stoe & Cie, 2013 ▸)
*T*_min_, *T*_max_	0.415, 0.615
No. of measured, independent and observed [*I* > 2σ(*I*)] reflections	7644, 1417, 1301
*R*_int_	0.045
(sin θ/λ)_max_ (Å^−1^)	0.685

Refinement	
*R*[*F* ^2^ > 2σ(*F* ^2^)], *wR*(*F* ^2^), *S*	0.017, 0.038, 1.06
No. of reflections	1417
No. of parameters	47
H-atom treatment	H-atom parameters constrained
Δρ_max_, Δρ_min_ (e Å^−3^)	0.34, −0.93

Computer programs: *X-AREA* and *X-RED32* (Stoe & Cie, 2013 ▸), *SHELXT* (Sheldrick, 2015*a* ▸), *SHELXL2014* (Sheldrick, 2015*b* ▸), *DIAMOND* (Brandenburg, 2006 ▸) and *publCIF* (Westrip, 2010 ▸).

## supplementary crystallographic information

### Crystal data

**Table:**

[Mn(C_2_H_6_OS)_6_]I_4_	*D*_x_ = 2.172 Mg m^−^^3^
*M**_r_* = 1031.31	Mo *K*α radiation, λ = 0.71073 Å
Trigonal, *R*3:*H*	Cell parameters from 10442 reflections
*a* = 11.8702 (10) Å	θ = 2.2–29.5°
*c* = 19.3860 (18) Å	µ = 4.75 mm^−^^1^
*V* = 2365.6 (5) Å^3^	*T* = 200 K
*Z* = 3	Block, brown
*F*(000) = 1467	0.16 × 0.12 × 0.05 mm

### Data collection

**Table:**

Stoe IPDS-2 diffractometer	1417 independent reflections
Radiation source: sealed X-ray tube, 12 x 0.4 mm long-fine focus	1301 reflections with *I* > 2σ(*I*)
Detector resolution: 6.67 pixels mm^-1^	*R*_int_ = 0.045
rotation method scans	θ_max_ = 29.2°, θ_min_ = 2.2°
Absorption correction: numerical (*X-RED32*; Stoe & Cie, 2013)	*h* = −16→16
*T*_min_ = 0.415, *T*_max_ = 0.615	*k* = −16→16
7644 measured reflections	*l* = −26→26

### Refinement

**Table:**

Refinement on *F*^2^	0 restraints
Least-squares matrix: full	Hydrogen site location: inferred from neighbouring sites
*R*[*F*^2^ > 2σ(*F*^2^)] = 0.017	H-atom parameters constrained
*wR*(*F*^2^) = 0.038	*w* = 1/[σ^2^(*F*_o_^2^) + (0.0105*P*)^2^ + 3.7009*P*] where *P* = (*F*_o_^2^ + 2*F*_c_^2^)/3
*S* = 1.06	(Δ/σ)_max_ = 0.001
1417 reflections	Δρ_max_ = 0.34 e Å^−^^3^
47 parameters	Δρ_min_ = −0.93 e Å^−^^3^

### Special details

**Table:**

Geometry. All esds (except the esd in the dihedral angle between two l.s. planes) are estimated using the full covariance matrix. The cell esds are taken into account individually in the estimation of esds in distances, angles and torsion angles; correlations between esds in cell parameters are only used when they are defined by crystal symmetry. An approximate (isotropic) treatment of cell esds is used for estimating esds involving l.s. planes.

### Fractional atomic coordinates and isotropic or equivalent isotropic displacement parameters (Å^2^)

**Table:**

	*x*	*y*	*z*	*U*_iso_*/*U*_eq_	
Mn1	0.3333	0.6667	0.6667	0.01931 (12)	
I1	0.6667	0.3333	0.75993 (2)	0.03170 (6)	
I2	0.6667	0.3333	0.58841 (2)	0.03067 (6)	
S1	0.54925 (4)	0.62436 (4)	0.58993 (2)	0.02341 (9)	
O1	0.50872 (12)	0.72442 (12)	0.60608 (6)	0.0247 (2)	
C1	0.67639 (18)	0.65612 (19)	0.64912 (11)	0.0325 (4)	
H1A	0.7149	0.6023	0.6373	0.049*	
H1B	0.6410	0.6353	0.6960	0.049*	
H1C	0.7433	0.7482	0.6466	0.049*	
C2	0.64415 (19)	0.6821 (2)	0.51353 (10)	0.0343 (4)	
H2A	0.6785	0.6249	0.5015	0.051*	
H2B	0.7165	0.7706	0.5212	0.051*	
H2C	0.5897	0.6829	0.4757	0.051*	

### Atomic displacement parameters (Å^2^)

**Table:**

	*U*^11^	*U*^22^	*U*^33^	*U*^12^	*U*^13^	*U*^23^
Mn1	0.01701 (16)	0.01701 (16)	0.0239 (3)	0.00851 (8)	0.000	0.000
I1	0.02446 (7)	0.02446 (7)	0.04619 (13)	0.01223 (4)	0.000	0.000
I2	0.03185 (8)	0.03185 (8)	0.02833 (10)	0.01592 (4)	0.000	0.000
S1	0.01918 (17)	0.02122 (18)	0.0279 (2)	0.00868 (15)	0.00044 (15)	−0.00439 (15)
O1	0.0219 (5)	0.0245 (6)	0.0294 (6)	0.0128 (5)	0.0038 (5)	0.0002 (5)
C1	0.0318 (9)	0.0368 (10)	0.0344 (9)	0.0212 (8)	−0.0054 (8)	−0.0033 (8)
C2	0.0298 (9)	0.0447 (11)	0.0283 (9)	0.0185 (9)	0.0040 (7)	−0.0047 (8)

### Geometric parameters (Å, º)

**Table:**

Mn1—O1^i^	2.1808 (12)	S1—C2	1.778 (2)
Mn1—O1^ii^	2.1808 (12)	S1—C1	1.7798 (19)
Mn1—O1^iii^	2.1808 (12)	C1—H1A	0.9800
Mn1—O1^iv^	2.1808 (12)	C1—H1B	0.9800
Mn1—O1^v^	2.1809 (12)	C1—H1C	0.9800
Mn1—O1	2.1808 (12)	C2—H2A	0.9800
I1—I1^vi^	2.8460 (5)	C2—H2B	0.9800
S1—O1	1.5204 (12)	C2—H2C	0.9800
			
O1^i^—Mn1—O1^ii^	180.00 (6)	O1—S1—C1	105.53 (9)
O1^i^—Mn1—O1^iii^	86.27 (4)	C2—S1—C1	98.56 (10)
O1^ii^—Mn1—O1^iii^	93.73 (4)	S1—O1—Mn1	119.38 (7)
O1^i^—Mn1—O1^iv^	93.73 (4)	S1—C1—H1A	109.5
O1^ii^—Mn1—O1^iv^	86.27 (4)	S1—C1—H1B	109.5
O1^iii^—Mn1—O1^iv^	180.00 (5)	H1A—C1—H1B	109.5
O1^i^—Mn1—O1^v^	93.73 (4)	S1—C1—H1C	109.5
O1^ii^—Mn1—O1^v^	86.27 (4)	H1A—C1—H1C	109.5
O1^iii^—Mn1—O1^v^	86.27 (5)	H1B—C1—H1C	109.5
O1^iv^—Mn1—O1^v^	93.72 (4)	S1—C2—H2A	109.5
O1^i^—Mn1—O1	86.28 (4)	S1—C2—H2B	109.5
O1^ii^—Mn1—O1	93.73 (4)	H2A—C2—H2B	109.5
O1^iii^—Mn1—O1	93.73 (4)	S1—C2—H2C	109.5
O1^iv^—Mn1—O1	86.27 (5)	H2A—C2—H2C	109.5
O1^v^—Mn1—O1	180.0	H2B—C2—H2C	109.5
O1—S1—C2	104.90 (9)		

Symmetry codes: (i) *y*−1/3, −*x*+*y*+1/3, −*z*+4/3; (ii) −*y*+1, *x*−*y*+1, *z*; (iii) −*x*+*y*, −*x*+1, *z*; (iv) *x*−*y*+2/3, *x*+1/3, −*z*+4/3; (v) −*x*+2/3, −*y*+4/3, −*z*+4/3; (vi) −*x*+4/3, −*y*+2/3, −*z*+5/3.

### Hydrogen-bond geometry (Å, º)

**Table:**

*D*—H···*A*	*D*—H	H···*A*	*D*···*A*	*D*—H···*A*
C1—H1*A*···I2	0.98	3.10	3.954 (2)	147
C2—H2*B*···I1^vii^	0.98	3.26	4.182 (2)	158
C2—H2*C*···I2^viii^	0.98	3.20	4.108 (2)	154

Symmetry codes: (vii) −*x*+5/3, −*y*+4/3, −*z*+4/3; (viii) −*x*+1, −*y*+1, −*z*+1.

## References

[bb1] Brandenburg, K. (2006). *DIAMOND*. Crystal Impact GbR, Bonn, Germany.

[bb2] Dubler, E. & Linowsky, L. (1975). *Helv. Chim. Acta*, **58**, 2604–2609.

[bb3] Garzón-Tovar, L., Duarte-Ruiz, Á. & Fanwick, P. E. (2013*b*). *Acta Cryst.* E**69**, m618. 10.1107/S1600536813028377 PMC3884268 24454044

[bb4] Garzón-Tovar, L., Duarte-Ruiz, A. & Wurst, K. (2013*a*). *Inorg. Chem. Commun.* **32**, 64–67.

[bb5] Glatz, M., Schroffenegger, M., Weil, M. & Kirchner, K. (2016). *Acta Cryst.* E**72**, 904–906. 10.1107/S2056989016008896 PMC4992903 27555928

[bb6] Grätzel, M. (2014). *Nat. Mater.* **13**, 838–842. 10.1038/nmat4065 25141800

[bb7] Groom, C. R., Bruno, I. J., Lightfoot, M. P. & Ward, S. C. (2016). *Acta Cryst.* B**72**, 171–179. 10.1107/S2052520616003954 PMC4822653 27048719

[bb8] Long, D.-L., Hu, H.-M., Chen, J.-T. & Huang, J.-S. (1999). *Acta Cryst.* C**55**, 339–341.

[bb9] Meek, D. W., Straub, D. K. & Drago, R. S. (1960). *J. Am. Chem. Soc.* **82**, 6013–6016.

[bb10] Sheldrick, G. M. (2015*a*). *Acta Cryst.* A**71**, 3–8.

[bb11] Sheldrick, G. M. (2015*b*). *Acta Cryst.* C**71**, 3–8.

[bb12] Stoe & Cie (2013). *X-AREA* and *X-RED32*. Stoe & Cie, Darmstadt, Germany.

[bb13] Stoumpos, C. C. & Kanatzidis, M. G. (2015). *Acc. Chem. Res.* **48**, 2791–2802. 10.1021/acs.accounts.5b00229 26350149

[bb14] Stranks, S. D. & Snaith, H. J. (2015). *Nature Nanotech*, **10**, 391–402. 10.1038/nnano.2015.90 25947963

[bb15] Tkachev, V. V., Lavrent’eva, E. A., Rosschupkina, O. S., Lavrent’ev, I. P. & Atovmyan, L. O. (1994). *Koord. Khim.* **20**, 674–676.

[bb16] Wang, K., Liang, Z., Wang, X. & Cui, X. (2015). *Adv. Electron. Mater.* **1**, 1500089.

[bb17] Westrip, S. P. (2010). *J. Appl. Cryst.* **43**, 920–925.

